# Alzheimer Disease Pathogenesis: Insights From Molecular and Cellular Biology Studies of Oligomeric Aβ and Tau Species

**DOI:** 10.3389/fnins.2019.00659

**Published:** 2019-06-21

**Authors:** Xu-Qiao Chen, William C. Mobley

**Affiliations:** Department of Neurosciences, University of California, San Diego, San Diego, CA, United States

**Keywords:** Alzheimer disease, amyloid plaques, neurofibrillary tangles, PET, Down syndrome, Aβ, tau, oligomer

## Abstract

Alzheimer disease (AD) represents an oncoming epidemic that without an effective treatment promises to exact extraordinary human and financial burdens. Studies of pathogenesis are essential for defining targets for discovering disease-modifying treatments. Past studies of AD neuropathology provided valuable, albeit limited, insights. Nevertheless, building on these findings, recent studies have provided an increasingly rich harvest of genetic, molecular and cellular data that are creating unprecedented opportunities to both understand and treat AD. Among the most significant are those documenting the presence within the AD brain of toxic oligomeric species of Aβ and tau. Existing data support the view that such species can propagate and spread within neural circuits. To place these findings in context we first review the genetics and neuropathology of AD, including AD in Down syndrome (AD-DS). We detail studies that support the existence of toxic oligomeric species while noting the significant unanswered questions concerning their precise structures, the means by which they spread and undergo amplification and how they induce neuronal dysfunction and degeneration. We conclude by offering a speculative synthesis for how oligomers of Aβ and tau initiate and drive pathogenesis. While 100 years after Alzheimer’s first report there is much still to learn about pathogenesis and the discovery of disease-modifying treatments, the application of new concepts and sophisticated new tools are poised to deliver important advances for combatting AD.

## Alzheimer’s Disease: Genetics and Neuropathology

### Genetics of AD

AD is the most common cause of dementia, accounting for up to 70% of cases (Plassman et al., 2007). Clinical manifestations, which are insidious in onset, include memory loss and cognitive decline as well as behavioral dysfunction and failure to maintain function in activities of daily living (Scheltens et al., 2016). Patients progress from normal cognition to Mild Cognitive Impairment (MCI) followed by increasing dementia severity (i.e., mild, moderate, and severe). It constitutes a leading cause of death; survival times average ∼8 years for those diagnosed at age 65 (Davis et al., 2018). Studies of AD pathogenesis are important for understanding the disorder and its treatment. Important sources of information include clinical presentation, genetics, neuropathology and cell biology. AD can be either sporadic or familial and of either late- or early-life onset. Age serves as the most important risk factor for AD. Sporadic AD is principally a disorder of the elderly, with most cases presenting after age 65; this form of the disease is referred to as late-onset AD (LOAD) or sporadic AD (SAD). Cases occurring at younger ages are referred to as early onset AD (EOAD). In some, but not all, EOAD cases there is a family history of dementia, allowing for the designation Familial AD (FAD). FAD cases are typically caused by mutations in the gene for the amyloid precursor protein (APP) or in the Presenilin genes, *PSEN1* and *PSEN2*, whose products participate in processing APP (see below for the steps in APP processing) (Bekris et al., 2010; Dorszewska et al., 2016). Another source of FAD are APP duplications (Kasuga et al., 2009; Shea et al., 2016). These cases support the view that increased expression of wild-type APP is sufficient for AD, a topic germane to the discussion of AD in Down syndrome (AD-DS), as will be discussed below. Further illustrating a role for APP are genetic evidence that the A673T substitution discovered in an Icelandic population is protective against AD as well as against cognitive decline in the elderly without AD (Jonsson et al., 2012). FAD cases usually present in the 40’s or 50’s. Distinct from FAD cases are those of early onset (i.e., age less than 65) in which there may be no family history and in which no definite genetic risk factor can be defined. Such cases are referred to as young onset AD (YOAD).

In addition to mutations that cause AD – i.e., those in *PSEN1*, *PSEN2*, and *APP*, a number of genetic factors have been discovered that confer increased risk of AD. Indeed, multiple variants have been discovered. The first such factor was the ε4 allele of *APOE*. The *APOE*ε4 allele has been unequivocally established as a potent risk factor (Corder et al., 1993; Bullido et al., 1998; Reitz and Mayeux, 2014). AD risk is reported to be increased by about 2 to 3 fold in those with one ε4 allele and by more than 10-fold in those with 2 alleles (Corder et al., 1993; Farrer et al., 1997; Kim et al., 2009; Bertram et al., 2010). Harboring one or two ε4 alleles is also linked to an earlier age of AD onset than those without an ε4 allele (van der Lee et al., 2018). A recent community-based cohort study in Rotterdam noted that the risk of AD at age 85 is 48% for those with two ε4 alleles, 18% with one ε4 allele, ∼9% with two ε3 alleles and only 5.5% with one or two ε2 alleles but no ε4 allele. Thus, the ε2 allele of *APOE* is associated with decreased risk of AD as well as with later age of onset (van der Lee et al., 2018). Studies of AD genetics, employing genome-wide association studies (GWAS), Whole Exome Sequencing (WES), and Whole Genome Sequencing (WGS) have defined additional genes whose variants contribute to increased risk. No fewer than 20 additional genes have been identified. The most consistently implicated are Clusterin (CLU), Sortilin-related receptor-1 (SORL1), ATP-binding cassette subfamily A member 7 (ABCA7), Bridging integrator 1 (BIN1), phosphatidylinositol binding clathrin assembly protein (PICALM), CD2 associated protein (CD2AP), Complement component (3b/4b) receptor 1 (CR1), CD33, triggering receptor expressed on myeloid cells 2 (TREM2), and phospholipase D3 (PLD3) (Karch and Goate, 2015). These variants define possible contributions in AD from genes that regulate endocytosis, inflammation and the brain’s innate immune system, and cholesterol/sterol metabolism (Karch and Goate, 2015; Selkoe and Hardy, 2016). While a role for recently discovered variants is evident, the increased risk attributed to each is far less than for the *APOE*ε4 allele (Strittmatter et al., 1993; Bertram et al., 2010; Genin et al., 2011; Karch and Goate, 2015; Di Battista et al., 2016). Importantly, the search for genetic risk variants as well as for protective variants continues. These studies promise to further inform understanding of AD pathogenesis and its treatment. In addition to genetic factors, environmental influences are recognized as conferring increased risk. Several factors including use of statins, light-to-moderate alcohol consumption, a Mediterranean diet, higher educational attainment, and physically and cognitively stimulating activities are associated with decreased risk. Increased risk of AD is correlated with age, head injury in males, diabetes, smoking, and lower social engagement (Hersi et al., 2017).

### Neuropathology of AD

#### Deposition of Aβ in Amyloid Plaques

**FIGURE 1:**
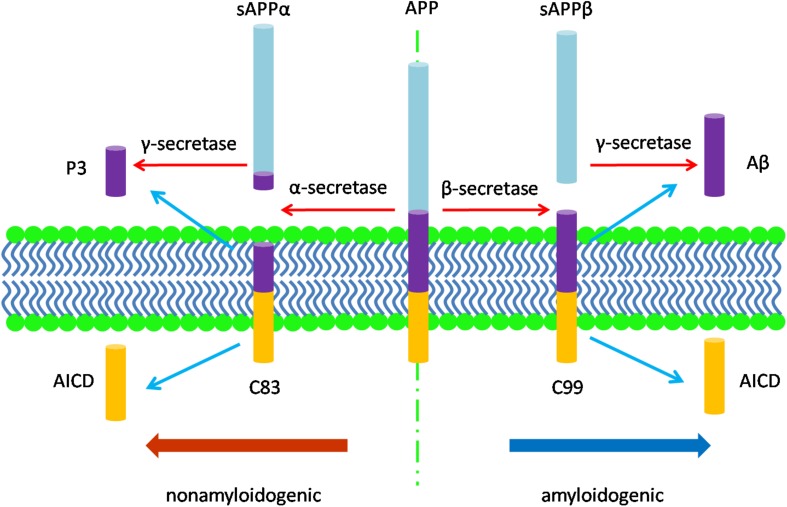
The diagram of APP processing. The type 1 transmembrane protein APP is processed by two pathways: the nonamyloidogenic pathway and the amyloidogenic pathway. In the nonamyloidogenic pathway, APP is cleaved by α-secretase to produce the soluble sAPPα and the C-terminal fragments, α-CTF (C83), which is then cleaved by γ-secretase to yield the APP intracellular domain (AICD) and P3 peptide; while in the amyloidogenic pathway, β-secretase cleaves APP to produce the soluble fragment sAPPβ and the C-terminal fragments, β-CTF (C99). C99 is then cleaved by γ-secretase to release AICD and Aβ peptides of varying length.

The neuropathology of AD manifests in several features. Macroscopically, there is brain shrinkage with cortical thinning and atrophy. First identified by Alzheimer, cardinal microscopic features include neuritic amyloid plaques and neurofibrillary tangles (NFTs). Amyloid plaques mark the extracellular accumulation and deposition of Aβ, a product of APP processing. APP, a type 1 transmembrane protein, is processed by sequential cleavage via either β- or α-secretase to produce the C-terminal fragments, β-CTF (i.e., C99) or α-CTF (i.e., C83), respectively. C99 is then cleaved by γ-secretase to yield the APP intracellular domain (AICD) and Aβ peptides of varying length; C83 cleavage yields the same AICD and the P3 peptide (Figure 1; Thinakaran and Koo, 2008). While APP processing leads to more Aβ40 than Aβ42, the latter is more amyloidogenic due to hydrophobic characteristics derived from the C-terminal alanine and isoleucine residues and this peptide is therefore more prone to aggregate (Burdick et al., 1992; Jarrett et al., 1993; Bitan et al., 2003) and to deposit in plaques. Aβ also accumulates around the cerebral vasculature, producing cerebral amyloid angiopathy (CAA) in which Aβ40 especially deposits in the vessel wall (Gravina et al., 1995; Serrano-Pozo et al., 2011a). Amyloid plaques appear to progress through stages, beginning with diffuse accumulation of extracellular Aβ followed by maturation to more dense deposits associated with a corona of dystrophic neurites (Selkoe and Hardy, 2016). An alternative view is that amyloid plaques are due to the aggregation of intracellular Aβ following the death of neurons (Gouras et al., 2000; D’Andrea et al., 2001; Pensalfini et al., 2014). Unlike diffuse plaques, dense-core deposits can be recognized by Thioflavin-S staining. Plaques are also immunopositive for antibodies against the Aβ peptide (Wisniewski et al., 1989). The neurites within plaques consist of both axons and dendrites (Su et al., 1993).

Amyloid deposition emerges regionally and in stages (Braak and Braak, 1991). Stage A is characterized by presence of amyloid deposits at low density, particularly in the basal portions of the frontal, temporal, and occipital lobes with sparing of hippocampus. In Stage B amyloid deposits of medium density are present in all but motor and sensory cortex; the hippocampus shows involvement, albeit mild, with occasional deposits in the pyramidal fields as well as dentate gyrus. Stage C shows increased involvement of the entire neocortex with continued presence of deposits in hippocampus and now with some subcortical regions involved, including striatum, thalamus, hypothalamus, cerebellum as well as the subthalamic and red nucleus (Braak and Braak, 1991). A later description of the temporal pattern for amyloid deposition defined five phases. The principal additional contributions were for: (1) at the time of hippocampal involvement (Phase 2 in this new scheme) involvement also of entorhinal, insular and cingulate cortex as well as amygdala; (2) in Phase 3, corresponding to Stage C, the presence of deposits in basal forebrain cholinergic nuclei at the time of involvement of other subcortical structures; (3) in Phase 4 the presence of Aβ deposits in the inferior olivary nucleus, the reticular formation of the medulla, substantia nigra, superior and inferior colliculus and red nucleus; and (4) in Phase 5, the additional involvement of the reticular formation of the pons, central and dorsal raphe, the locus coeruleus, and other pontine nuclei as well as the molecular layer of the cerebellum (Thal et al., 2002). While amyloid deposition serves as a cardinal neuropathological feature, amyloid burden, whether measured as total plaques or as dense and neurite-containing plaques, fails to correlate with severity or duration of dementia (Arriagada et al., 1992; Bierer et al., 1995; Giannakopoulos et al., 2003). Thus, while Aβ deposition features prominently in AD clinico-pathological studies fail to support a defining role for plaque deposits in its onset or temporal progression.

#### Deposition of Tau and Phosphorylated Tau: Pre-tangles and NFTs

In contrast to the lack of correspondence between plaque burden and dementia, the case for a link between NFTs and disease progression is stronger. Indeed, the amount and distribution of NFTs has been shown to correlate with both the severity and duration of dementia (Arriagada et al., 1992; Bierer et al., 1995; Gomez-Isla et al., 1997; Giannakopoulos et al., 2003). Aberrantly folded and abnormally phosphorylated isoforms of the microtubule-associated protein tau serve as the principle constituent of NFTs (Grundke-Iqbal et al., 1986; Kosik et al., 1986; Wood et al., 1986). NFTs are present initially and principally intracellularly. Neuropil threads are aggregates of tau and phosphorylated tau (p-tau) located in neuronal processes; they are present together with NFTs in the AD brain (Perry et al., 1991; Binder et al., 2005). Based on morphological observations, NFTs can be subdivided into three stages (Braak et al., 1994; Augustinack et al., 2002): (1) diffuse, occasional punctate and non-fibrillar pre-NFTs, or pre-tangles: recognized by antibodies against tau phospho-epitopes pT153, pS262, and pT231 as well as by antibodies against conformational epitopes, including those detected by antibodies MC1, T22, and Alz50 (Hyman et al., 1988; Jicha et al., 1997; Augustinack et al., 2002; Lasagna-Reeves et al., 2012b); (2) mature intracellular cytosolic NFTs which consist of filamentous aggregates of tau and phosphorylated tau: recognized by silver and Thioflavin-S staining; p-tau antibodies AT8, AT100, PHF1, pT175/181, 12E8 (pS262/pS356), pS422, pS46, pS214; and the Alz50 epitope (Arnold et al., 1991; Reusche, 1991; Augustinack et al., 2002); and (3) extraneuronal “ghost” NFTs, presumably representing tangles once present in neurons that have died: recognized by silver and Thioflavin-S; AT8, AT100, and PHF1 (Arnold et al., 1991; Reusche, 1991; Augustinack et al., 2002). Deposition of NFTs follows a stereotypical spatiotemporal pattern. Braak and Braak defined six stages in the evolution of NFT involvement of the dementing brain (Braak and Braak, 1991). The overall pattern is characterized by initial involvement of medial temporal cortex with later involvement of most of isocortex (i.e., neocortex), but with relative sparing of primary sensory, motor, and visual cortex. Stages I and II feature initial involvement of transentorhinal and entorhinal cortex and define the “transentorhinal stage” of disease. Stage II is characterized by progressive involvement of these regions together with inclusion of hippocampal CA1 region, temporal cortex as well as magnocellular forebrain nuclei and anterior dorsal nucleus of the thalamus. Stage III sees progressive involvement of the areas impacted by Stage II as well as mild involvement of subiculum, the additional hippocampal subfields, amygdala, reuniens nucleus of thalamus and tuberomammillary nucleus. In Stage IV the same regions show progressive involvement with mild involvement of claustrum and the first changes in neocortex. Stages III and IV have been labeled the “limbic stage” of disease. In Stage V the changes in limbic cortex just referenced are more severe with dense accumulations of NFTs in transentorhinal and entorhinal cortex, temporal isocortex, essentially all hippocampal regions and subiculum and presubiculum involved. In Stage V for the first time there is marked involvement for essentially all areas of association neocortex with relative sparing of primary sensory and motor regions. The changes in Stage VI are more severe in all affected regions with continued relative sparing of motor and sensory neocortex but with NFTs now appearing in medium-sized neurons of the striatum and neurons of the substantia nigra. Stages V and VI thus constitute the “isocortical stage” of the disease.

A recent study attempted to define the earliest stage of accumulation of p-tau in an autopsy series of more than 2,000 individuals aged 1 to 100 years. Age-related changes defined a series of pre-tangle stages in which p-tau was found first in the proximal axons of neurons of the locus coeruleus in the brains of young people. Apparent progression of lesions resulted in involvement of the somatodendritic compartment of these cells and then their presence in neurons of the raphe system and magnocellular nuclei of the basal forebrain. Interestingly, in this early pre-tangle stage of pathology p-tau accumulation did not involve cortex. Subsequent pre-tangle stages did involve cortex with preferential involvement of transentorhinal cortex (Braak et al., 2011). These findings raise the possibility that involvement of locus coeruleus and projection neurons of the raphe and basal forebrain are impacted early in the emergence of AD-related pathology with later involvement of cortical structures. The findings of Mesulam and Colleagues support the early appearance of tangle and pre-tangle pathology in basal forebrain cholinergic neurons (BFCNs) in the nucleus basalis of Meynert (NBM) (Mesulam et al., 2004). Note, however, that a recent study argues for initiation of tau pathology in the transentorhinal/entorhinal region (Kaufman et al., 2018). It is tempting to infer that a continuum exists between pre-tangle and tangle pathology and between the earliest pattern of involvement and that associated with AD. However, the cross-sectional nature of autopsy studies, and the absence of symptoms in pre-tangle stages, limits the ability to conclude this. Nevertheless, they point to the possible emergence of AD pathology in a select number of cortically projecting neurons decades before dementia ensues.

#### PET Imaging to Detect Aβ and Tau Pathologies

A significant advance in defining AD-related pathology, and thereby for enabling both cross-sectional and longitudinal studies, has been the development of positron emission tomography (PET). The advent of specific amyloid-β PET tracers enabled the non-invasive detection of amyloid neuritic plaques in subjects. The first such reagent, Pittsburgh compound B (PiB), a radioactive analog (^11^C) of Thioflavin-T, selectively binds to fibrillar amyloid, demonstrates good sensitivity and is widely used to image amyloid plaques in PET studies of AD. Close to 90% of AD cases display PiB-PET positivity, which correlates with the plaque distribution, but correlates only moderately with neurodegeneration and cognitive deficits (Dubois et al., 2010, 2014; Laforce et al., 2018). However, there are disadvantages to the use of ^11^C-labeled tracers: a radioactive decay half-life of about 20 min and scan time limited to about 30 min. Thus, only centers with essentially immediate access to cyclotrons are able to employ ^11^C-labeled tracers (Yeo et al., 2015). Second generation amyloid-β PET ligands, which employ the longer lived fluorine 18–labeled (^18^F) as the radioactive atom, show excellent sensitivity and specificity and perform comparably to PiB (Filippi et al., 2018). Three novel ^18^F tracers, ^18^F-florbetapir, ^18^F-florbetaben, and ^18^F-flutemetamol (Wong et al., 2010; Villemagne et al., 2012; Rominger et al., 2013; Curtis et al., 2015) have extended the ability to use PET to monitor disease progression and amyloid accumulation. Given the temporal and spatial correlation of tau pathology with clinical manifestations in AD, tau was an obvious choice for targeting with PET tracers. All available tau tracers, including flortaucipir, display appreciable access to brain and bind to paired helical filaments without appreciable binding to amyloid-β deposits. Importantly, tau PET tracer binding recapitulates the topographical distribution of Braak NFT pathology and thus correlates with clinical symptoms and neurodegeneration in AD. Combining amyloid-β and tau PET is expected to provide benefits for evaluating the emergence of these pathologies in AD (Laforce et al., 2018). The development of additional PET ligands, including those that bind to the earliest molecular assemblies that mark AD pathogenesis, promises to be even more useful for detecting AD (see below). Finally, the metabolic consequences of AD have been pursued using ^18^F-fluorodeoxyglucose (^18^F-FDG)-PET. Uptake of labeled FDG allows for estimation of neuronal activity. In AD this method demonstrates glucose hypometabolism in the parietotemporal association cortex, posterior cingulate cortex, precuneus and, as disease progresses, in frontal cortex (Marcus et al., 2014).

#### Synaptic Deficits and Neuron Loss

Though less well characterized than the plaque and tangle, the events most important to onset and progression in AD are likely to be early deficits in synaptic function with later loss of neurons (Ball, 1977; Whitehouse et al., 1981; DeKosky and Scheff, 1990; Scheff et al., 1990, 2006, 2007; Scheff and Price, 1993; Gómez-Isla et al., 1996; Serrano-Pozo et al., 2011a; Andrade-Moraes et al., 2013; Calderon-Garciduenas and Duyckaerts, 2017). Both synapse loss and neuronal loss contribute to macroscopic cortical atrophy. Using synaptic protein markers, synaptic loss was documented in the dentate gyrus in early AD (Hamos et al., 1989; Honer et al., 1992; Masliah, 1995). Electron microscopic analysis of CA1 hippocampus showed loss of synapses in both MCI and mild AD and these changes were correlated with cognitive dysfunction, but not Braak stage (Scheff et al., 2007). A statistically significant reduction in total synapses together with a decrease in volume were seen in the outer molecular layer (OML) of the dentate gyrus in early AD (mean Mini-Mental State Examination (MMSE) = 18.6). There was also a decrease (13%) in synaptic number in this region in MCI (mean MMSE = 26.8), but the difference did not reach statistical significance. In examining the OML for total synapse number and volume across early AD, MCI and cognitively normal subjects (mean MMSE for normal subjects = 28.1) there were statistically significant positive correlations with MMSE and certain other tests of cognition. Given innervation of OML by layer II of the entorhinal cortex, a significant negative correlation was also detected between synapse number in the OML and the density of NFTs in this region. However, there was no such correlation with Braak stage (Scheff et al., 2006). These data are evidence for a loss of synapse forming entorhinal cortex afferents that is correlated with cognitive dysfunction. Given that loss of layer II neurons has been documented in those with MCI (a decrease of 57 to 63% versus cognitively normal controls), degeneration of layer II neuron likely contributes to the decrease in OML afferents (Gómez-Isla et al., 1996; Kordower et al., 2001). Synaptic loss was also demonstrated in the neocortex of those with AD examined at the time of biopsy or at post-mortem exam; synapse loss was correlated with cognitive dysfunction (DeKosky and Scheff, 1990). Additional studies to explore regional and temporal characteristics of synapse loss could help to further define this aspect of neuropathology. Neuronal loss postdates synaptic dysfunction. The earliest phase of neuron loss impacts entorhinal cortex, locus coeruleus and the NBM. At later stages all three populations show progressive neuron loss with cell loss most pronounced in the NBM (Arendt et al., 2015). A recent study determined neuronal number in hippocampus, cortex and subcortical structures in elderly females. Controls showed no amyloid plaques, were Braak Stage II or less, and had no history of cognitive or behavioral deficits. A second group had AD-related pathology with significant amyloid plaque pathology and were Braak Stage IV or greater, but also had no history of behavioral or cognitive deficits. A third group consisted of patients with a clinical diagnosis of dementia (CDR = 3), showed significant amyloid plaque deposition and were Braak Stage III or greater. Comparing those with AD pathology and dementia to those with pathology but not dementia, there was robust reduction in the number of neurons in hippocampus, cortex and subcortical regions (including basal forebrain nuclei, diencephalon, and brainstem) together with an increase of non-neuronal cells in cortical gray and subcortical white matter. Remarkably, comparing those with AD pathology without dementia and controls, there was no significant difference in neuron number. Given the preservation of neuron number and cognition in spite of amyloid plaques and NFTs, the data are evidence that neuron loss is more closely linked to cognitive dysfunction than the classical hallmarks of AD neuropathology (Andrade-Moraes et al., 2013). Combined with data for correlation of synapse loss with disease progression, they suggest that a focus on synapse and neuron loss will be essential for deciphering the pathogenesis of AD.

#### Other AD Pathological Hallmarks

Changes in early endosomes are found in AD [reviewed in Chen et al. (2018)]. Enlargement of early endosomes, a phenotype consistent with excessive activation of Rab5 (Roberts et al., 1999), is found in neurons in the brains of those with both sporadic and familial AD (Cataldo et al., 1997, 2000, 2001). In early stage AD (corresponding to Braak Stages I-III with sparse amyloid plaques) enlarged early endosomes were present in most neurons in layer II of entorhinal cortex, in the CA2 and CA3 subfields of hippocampus and in the prefrontal cortex in layers III and V. Thus, the size of early endosomes was impacted long before extensive amyloid deposits and NFTs (Cataldo et al., 2000). Interestingly, in early AD enlargement of early endosomes was exacerbated by the *APOE*ε4 allele, suggesting a link between *APOE*ε4 and early endosomes (Cataldo et al., 2000). Endosomal phenotypes also impact many neurons in the brain of a segmental trisomy 16 mouse model of DS, the Ts65Dn mouse, including neurons in neocortex, hippocampus as well as the medial septal nucleus (MSN), and NBM of basal forebrain (Cataldo et al., 2003). Endosome enlargement coincides with increasing levels of Aβ peptide during AD progression and importantly, intracellular Aβ is present in enlarged Rab5-labeled early endosomes, suggesting that Aβ may directly contribute to dysregulation of the endosomal system (Cataldo et al., 2004).

Other classical neuropathological hallmarks of AD are granulovacuolar degeneration (GVD) and Hirano Bodies (Ball, 1977, 1978; Xu et al., 1992; Hirano, 1994). The former consist of double-membrane bodies of uncertain significance but demonstrate the presence of markers of late stage autophagy, including LAMP1 (lysosomal-associated membrane protein 1) and cathepsin D, suggesting that this is a late stage autophagosome. Given colocalization of CHMP2B (charged multivesicular body protein 2B) with GVBs this organelle may represent a point of intersection between the endocytic and autophagic pathways (Funk et al., 2011). Hirano bodies are refractile eosinophilic rod-like structures that contain a number of components, including actin and other cytoskeletal proteins (Goldman, 1983; Galloway et al., 1987). How they arise and their contribution, if any, to pathogenesis is poorly understood. Activation of astrocytes and microglial cells is also seen in AD and both are associated with dense-core amyloid plaques. Astrocytosis and microgliosis increase linearly throughout the disease course. Glial responses correlated positively with tangle burden suggesting linkage between glial responses and neurofibrillary pathology (Serrano-Pozo et al., 2011b). Considerable recent interest has focused on a role for activation of microglia, the resident immune cells in brain, in mediating AD pathogenesis. In early stage AD microglia are believed to respond to Aβ by becoming activated and migrating to plaques to phagocytose Aβ, thus enhancing clearance of Aβ levels. With sustained microglial activation, however, exacerbation of AD may result from a combination of defective Aβ phagocytosis and release of proinflammatory cytokines. The net effect is that activated microglia could contribute to neuronal injury (Kinney et al., 2018). These findings combine with others, including evidence for activation of the complement system in the AD brain, to underpin the “inflammatory hypothesis” of AD (McGeer et al., 2016).

#### Biomarkers of AD in CSF and Blood

AD pathological features have inspired the development of biomarkers to assist in defining the diagnosis and progression of AD. The core cerebrospinal fluid (CSF) biomarkers in AD and prodromal AD include increased p-tau and total tau together with decreased Aβ42 (Olsson et al., 2016). Recent studies in humans point to increased levels of total tau as due to increased synthesis and release; the increase is positively correlated with amyloidosis (Sato et al., 2018). The increase in p-tau may be a consequence of increased total tau as well as disease-related increases in the production of phosphorylated tau species. The decrease in Aβ42, and in the Aβ42/Aβ40 ratio, reflects increased deposition of this Aβ species in the brain of those with amyloid plaques (Potter et al., 2013). A blood-based biomarker of Aβ kinetics would satisfy the very great need for noninvasively assessing the status of brain amyloid deposition. Recent studies point to a reduced Aβ42/Aβ40 ratio in plasma in amyloid PET-positive subjects as satisfying this need (Fandos et al., 2017; Ovod et al., 2017). The importance attached to synaptic loss has motivated attempts to discover relevant biomarkers. One such biomarker is neurogranin, a dendritic protein whose increased CSF levels reflect dendritic instability in prodromal AD and AD (Kvartsberg et al., 2015a, 2015b). Another promising marker is the neurofilament light (NFL) protein, a neurofilament component present in axons where it along with microtubules and actin constitutes the neuronal cytoskeleton (Yuan et al., 2012). Presumably marking axonal injury and loss, increased levels of NFL are present in the CSF and plasma in MCI and AD (Mattsson et al., 2017). Importantly, the levels of NFL in CSF and plasma are tightly correlated (Mattsson et al., 2017), thus offering the ability to assess progression of axonal loss. It is noteworthy, however, that increased NFL is not specific to AD, serving as a biomarker for other causes of neurodegeneration (Blennow, 2017). While there is now an armamentarium of biomarkers for predicting and detecting AD, the need for additional biomarkers sensitive to early synaptic damage and dysfunction remains.

## Alzheimer Disease in Down Syndrome: Neuropathology, Genetics, and Cell Biology

Down syndrome (DS), the most common genetic cause of AD, may offer unique insights into AD pathogenesis. DS is due to trisomy for all or part of a third copy of chromosome 21. It is associated with the presence of a number of abnormal clinical phenotypes, including changes in craniofacial anatomy, and is universally characterized by mild to moderate intellectual disability. The clinical presentation of dementia follows a course similar to AD, with problems with recall, explicit memory, and receptive language function before frank dementia. In those aged 30 to 40 the process may feature changes in behavior and personality (Ballard et al., 2016). Almost all adults with DS develop AD-like neuropathology by the age of 40. More than 50% display progressive cognitive impairment. The mean age of onset of dementia is 55. Some estimates are that greater than 80% suffer with dementia beyond age 65 (Hithersay et al., 2017). Given these findings this disorder is now termed AD in DS (AD-DS). DS is of particular interest in the context of AD because it defines a very large at-risk population in which longitudinal studies of pathogenesis and disease progression could inform the understanding of AD more generally and enable studies of treatments to prevent AD in those with DS.

Among the genes on chromosome 21 triplicated in DS the evidence is compelling that increased gene dose for *APP*, with increased levels of the APP protein and its products, is necessary for AD-DS (Prasher et al., 1998; Korbel et al., 2009; Wiseman et al., 2015; Ballard et al., 2016; Doran et al., 2017). Other chromosome 21 genes may impact AD in DS, including *DYRK1A*, a kinase that acts on tau and APP and other substrates possibly relevant for AD pathogenesis, and *SOD1*, an important regulator of reactive oxygen species (Wiseman et al., 2015). Variants of genes not on chromosome 21 also contribute. Indeed, published studies support *APOE*ε4 as a risk factor for the development of dementia in DS (Schupf et al., 1996; Prasher et al., 2008). Other AD risk variants including SORL1 and PICALM also contribute to the emergence of AD in DS (Hyman et al., 1995; Lee et al., 2007; Jones et al., 2013).

Given the APP gene dose dependence of AD in DS, and the recognition that a rare form of FAD is due to APP gene duplication, it is not surprising that AD and AD-DS share common neuropathologies, including amyloid plaques and NFTs (Rafii, 2018). Indeed, it appears that the neuropathological hallmarks of AD-DS are qualitatively very similar if not identical to AD, albeit with some quantitative changes with respect to lesion density in certain brain regions (Hof et al., 1995; Hyman et al., 1995; Davidson et al., 2018). Thus, like AD, Aβ – principally Aβ42 – is the Aβ isoform present in diffuse deposits and mature plaques; Aβ40 accumulates around cerebral vessels, thus recapitulating the CAA of AD, but shows a significantly higher deposition in DS than in AD (Head et al., 2017). As might be predicted, Aβ accumulation in DS occurs in young people with deposition in diffuse plaques as early as the teenage years – i.e., decades earlier than in aged controls and AD. Essentially all those patients above age 20 showed Aβ deposits and these were present across the neocortex as well as hippocampus; they were somewhat less numerous in hippocampus than neocortex in younger patients. As in AD, amyloid deposition is well established before NFT formation (Hof et al., 1995) which begins in the late 30 s in the entorhinal cortex and increases into the 40 s and 50 s at which time NFTs are present in hippocampus and neocortex (Hof et al., 1995; Hyman et al., 1995; Wiseman et al., 2015; Ballard et al., 2016). Also as in AD, NFTs correlate more closely with dementia than does Aβ deposition.

*In vivo* PET imaging has been used to assess amyloid and tau accumulation in people with DS. Unlike histological findings, PET imaging shows that the earliest site of Aβ accumulation in DS is striatum with later increases registered in frontal, parietal, temporal, and anterior cingulate cortex (Annus et al., 2016). In nondemented DS adults, PET amyloid positive subjects examined at an interval of 3 years showed a statistically significant loss of gray matter volume in parietal cortex and precuneus but no significant change in cognitive function (Lao et al., 2017). In a recently published study of a small number of subjects, amyloid PET positivity was found to predate tau PET positivity (Rafii et al., 2017). Tau accumulation by PET in adults with DS showed binding similar to that AD, including involvement of medial temporal, inferolateral temporal, precuneus, and posterior cingulate regions (Rafii et al., 2017). Tau binding correlated with subject age and with amyloid PET score. Increasing tau binding was negatively correlated with cognitive performance and positively correlated with AD-like volume loss (Rafii et al., 2017).

Synaptic loss is not well studied in DS. Neuronal loss is evident early in entorhinal cortex and subcortical areas of DS brain, including locus coeruleus and basal forebrain nuclei. Neuronal loss later involves hippocampus and temporal cortex and other cortical areas (Mann et al., 1987; Head et al., 2016). At the gross anatomical level, progressive hippocampal atrophy results in enlargement of lateral ventricles (Mann et al., 1990; Hof et al., 1995). Dysregulation of the endosomal pathway is a striking feature of AD-DS. Enlargement of early endosomes and increased Rab5 activation was present decades before formation of amyloid plaques and NFTs (Chen et al., 2018). Indeed, enlargement of early endosomes has been documented in fetal brain (Cataldo et al., 2000). Remarkably, as in AD, early endosomes were found to contain Aβ (Cataldo et al., 2004). AD-concurrent pathologies, including GVD and Hirano Bodies, are also present in individuals with DS (Ellis et al., 1974; Ball and Nuttall, 1980). Among the biomarkers in AD, plasma NFL and CSF biomarkers including total tau, p-tau and NFL have good diagnostic performance to detect AD in DS (Fortea et al., 2018).

## Alzheimer’s Disease: Molecular and Cell Biology

### Evidence for Toxic Soluble Aβ Oligomers in Pathogenesis

It is increasingly apparent that AD pathogenesis is more tightly linked to toxic assemblies of the proteins that contribute to amyloid plaques and NFTs than to these structures themselves. Indeed, oligomeric complexes of Aβ and tau are now thought to confer disease-relevant toxic actions. In the case of Aβ, the “Aβ oligomer hypothesis” has supplanted the “amyloid cascade hypothesis”; proposed more than 25 years ago, this theoretical construct that did much to guide research on AD (Selkoe, 1991; Hardy and Higgins, 1992; Selkoe and Hardy, 2016). Recent reviews detailing the emergence of the Aβ oligomer (AβO) hypothesis provide a wealth of insights (Selkoe and Hardy, 2016; Cline et al., 2018). Several findings point to AβOs rather than amyloid plaques as playing a central role in pathogenesis: (1) synaptic loss was present in APP transgenic models of AD in the absence of plaque formation (Mucke et al., 2000); (2) in the absence of plaques, intraneuronal Aβ was correlated with cognitive dysfunction in several APP transgenic models (Billings et al., 2005; Knobloch et al., 2007; Leon et al., 2010; Iulita et al., 2014); (3) in a mutant APP transgenic rat model, as well as in a small sample of human AD subjects, intracellular Aβ was present in Reelin-positive neurons in layer II neurons of the entorhinal cortex, including the pre-plaque stage in rats and at Braak stage I in humans (Kobro-Flatmoen et al., 2016); (4) AβOs showed an age-related increase in the brains of APP transgenic mice with accompanying reductions in the levels of synaptic proteins (Pham et al., 2010); (5) antibodies to AβOs reversed deficits in hippocampally mediated cognition in the mouse models of AD (Billings et al., 2005; Xiao et al., 2013); (6) punctate intracellular Aβ immunostaining, presumably AβOs, were present in the brains of children with DS in the absence of plaques more than a decade before plaques were detected (Gyure et al., 2001); (7) AβOs were increased in the AD brain with respect to controls and were correlated with severity of cognitive impairment and loss of synaptic markers (Lue et al., 1999; McLean et al., 1999; Naslund et al., 2000; Steinerman et al., 2008; Pham et al., 2010; Lesne et al., 2013; Cline et al., 2018); (8) using a highly sensitive assay, increased levels of AβOs were present in CSF of AD patients (Georganopoulou et al., 2005); and (9) in cases with equal amyloid plaque burden the levels of AβOs differentiated between those with versus without cognitive deficits (Esparza et al., 2013).

That AβOs exert toxic activity is well established (Cline et al., 2018). In an early, robust demonstration from the Walsh and Selkoe labs, AβOs extracted from AD brain reduced long-term potentiation (LTP) and increased long-term depression (LTD) in mouse hippocampal slices. The effects were due to Aβ because they were blocked with extracts immunoprecipitated with antibodies to Aβ, including antibodies to the amino-terminus of the peptide. Partially purified AβO-containing extracts also reduced spine density in organotypic rat hippocampal slices. AβO effects on LTD were mediated by the metabotropic glutamate receptors (mGluRs) but not the N-methyl-D-aspartate receptors (NMDARs), while the latter did mediate the effect on spine density. In addition, AβO-containing extracts injected into the lateral ventricle of rats impaired recall of a learned behavior (Shankar et al., 2008). Remarkably, while AβOs led to toxic activity, soaking amyloid plaque cores in physiologic buffer failed to result in a supernatant with LTP blocking activity. In addition to these findings, many additional toxic activities have been attributed to AβOs including induction of tau pathology, insulin resistance, impaired axonal transport, loss of expression of trophic factors, and neuronal death [reviewed in Cline et al. (2018)].

Important questions remain as to the structure of toxic AβOs and how they induce their effects. Regarding the former, the evidence is clear that AβOs present in a soluble form are toxic. Indeed, in recent studies the Walsh lab compared the toxic AβO activity in AD cortical homogenates versus soluble extracts (Hong et al., 2018). The former were prepared by homogenizing AD cortical tissue in artificial CSF followed by collecting the 200,000g supernatant. The latter were collected when small chunks of AD cortical gray matter were soaked for 30 min at 4°C in artificial CSF devoid of detergents. The most salient finding was that Aβ-containing toxic species were significantly enriched in the soluble AβO collection. AβOs readily diffused into the buffer soaking brain chunks and eluted from size-exclusion chromatography in fractions representing a broad distribution of molecular sizes, ranging from the column exclusion volume (i.e., >700 kDa) to intermediate molecular sizes (i.e., ∼150 kDa) to low molecular sizes (∼17 kDa). Relative to the supernatants of cortical homogenates, soluble AβOs contained less Aβ in the high molecular size fractions and more in the intermediate and low molecular size fractions. The specific activity of soluble AβOs in supernatants and homogenates were compared in studies of induced pluripotent stem cells (iPSC)-derived human neurons in which live imaging was used to assess impact neurite length. A time-dependent decrease was demonstrated for AβOs in both sources; activity was blocked by immunodepleting samples with antibodies to Aβ. Significantly, though the amount of Aβ present in the soluble AβO preparation was less than in the homogenate-derived preparation (∼8-fold difference in Aβx-42), the specific activity was considerably increased. Studies of AβO effects in blocking LTP showed the same findings; soluble AβOs blocked LTP induction with a specific activity greater than AβOs in the homogenate-derived preparation. These data are evidence that toxic activity due to AβOs is largely present in a soluble diffusible species and that toxic species may represent a minority of soluble AβO and Aβ species in AD brain (Hong et al., 2018).

While the solubility of such species is established, and their content of Aβ is clear, there is no agreement on the size, Aβ peptide types present, or conformations that contribute to toxicity. Evidence has been amassed to support the presence of toxic species in both high molecular weight (HMW) and low molecular weight complexes, including impressive support for the activity of Aβ dimers (Shankar et al., 2008; Cline et al., 2018). In a recent step toward creating reagents that support further definition of the structure of toxic AβO species, the Walsh lab has shown that an aggregate-preferring murine monoclonal Aβ antibody, 1C22, acts potently to prevent neurite retraction and accumulation of p-tau in cultured human neurons exposed to soluble AβOs, prepared as homogenates, essentially as described above (Jin et al., 2018). 1C22 potency exceeded that of other selected anti-Aβ antibodies tested. Indeed, at a concentration of Aβx-42 of 1.55 nM, the IC50 (the half-maximum inhibitory concentration) for blocking neurite retraction by 1C22 was estimated to be 5.33 nM – i.e., only a four-fold excess over the soluble AβO level. These studies point to the utility of antibody-related approaches for defining the structure(s) of toxic AβO species and for defining potentially effective treatments to protect against AβOs (Jin et al., 2018). A recent report points to an alternative method for isolating biologically active soluble Aβ-containing species from the AD brain. Added to HEK293T cells transfected with a fluorescently tagged Aβ42 construct expressed in cytosol, this preparation induced aggregation of the construct. Importantly, it did not induce aggregation of a corresponding fluorescently tagged α-synuclein construct. The findings point to the presence within this preparation of a prion-like activity directed against Aβ42 [(Aoyagi et al., 2019); also see below].

How AβOs exert their toxic activity is another area of great interest. Evidence to support a role for cell surface receptors is compelling and a diverse and relatively large number of molecules have been identified as candidates, among which are ionotrophic and metabotropic glutamate receptors, ephrin receptors, receptor tyrosine kinases, immunoglobulin and immunoglobulin-like receptors as well as cell adhesion molecules and heparin sulfate proteoglycans (HSPGs) (Jarosz-Griffiths et al., 2016; Cline et al., 2018; Mroczko et al., 2018). The list includes cellular prion protein (PrP^C^), Na^+^/K^+^ ATPase alpha 3 subunit (NKAα3), mGluR5, NMDARs, the α-amino-3-hydroxy-5-methyl-4-isoxazolepropionicacid receptors (AMPARs), frizzled, neuroligin 1, neurexin 2α, β2-adrenergic receptor (β2-AR), α7-nicotinic acetylcholine receptor (α7nAChR), insulin receptors, p75NTR, ephrin type A receptor 4 and B receptor 2 (Eph4A and EphB2), leukocyte immunoglobulin-like receptor subfamily B member 2 (LilrB2), and the receptor for advanced glycation end products (RAGE), among others (Table 1). The diversity of binding receptors may be a product of the inherent hydrophobicity of AβOs or may be due to conformational states that support specific receptor binding. In any case, downstream signaling events can be envisioned to be impacted by AβO binding leading to up- or down-regulation of their respective pathways. One such event could be dysregulation of calcium homeostasis (Alzheimer’s Association Calcium Hypothesis Workgroup, 2017). Future studies of AβO-mediated toxicity will benefit from the ability to define toxic species, identify their specific receptors, and decipher the contributions of downstream signaling events that contribute to toxic actions.

**TABLE 1:** Summary of receptor proteins involved in binding of Aβ species.

**Receptors**	**Aβ species**	**References**
PrP^C^	Oligomer	Lauren et al., 2009; Chen et al., 2010; Gimbel et al., 2010; Dohler et al.,2014
NKAα3	Oligomer	Ohnishi et al., 2015; DiChiara et al.,2017
mGluR5	Oligomer	Um et al.,2013
NMDARs	Oligomer	De Felice et al., 2007; Lacor et al.,2007
AMPARs	Oligomer	Zhao et al.,2010
frizzled	Oligomer	Magdesian et al.,2008
neuroligin 1	Oligomer	Brito-Moreira et al.,2017
neurexin 2α	Oligomer	Brito-Moreira et al.,2017
β2-AR	Dimer	Wang et al.,2010
α7nAChR	Oligomer	Wang et al.,2000a, b
insulin receptors	Oligomer	De Felice et al.,2009
p75NTR	Oligomer	Yaar et al., 1997, 2002; Knowles et al.,2009
Eph4A and EphB2	Oligomer	Lacor et al., 2007; Cisse et al., 2011; Vargas et al.,2014
LilrB2	Oligomer	Kim et al.,2013
RAGE	Oligomer	Yan et al., 1996; Deane et al., 2003; Sturchler et al.,2008

The presence of AβOs in the extracellular space raises the possibility of cell-to-cell spread. Such “spread” might explain the temporal pattern of increasing amyloid deposition by allowing for a focus of AβO to move from one brain region to the next, possibly on the basis of synaptic connectivity. The additional possibility exists that spread-mediated transport of AβOs, including toxic isoforms, would serve as templates or seeds to induce structural conversion of Aβ species, thus increasing AβOs and amyloid deposits at the sites of spread. The latter, termed propagation, suggests the possibility of templated conversion of normal to abnormal structural isoforms, as is seen in prion diseases. Prions are defined as alternatively folded self-propagating protein conformers (Sigurdson et al., 2018). Gouras and colleagues provided evidence for prion-like behavior of Aβ species in studies using N2A cells expressing the Swedish APP mutation (N2A:APP_Swe_). Extracts from either the APP/PS1 mouse brain or the brains of Tg19959 mice that express the Swedish and London APP mutations, but not wild type mouse brain, added to N2A:APP_Swe_ cells resulted in clonal lines containing puncta immunopositive for antibodies that detect AβOs and/or Aβ fibrils. Remarkably, after repeated passaging positive clones continued to show such puncta. Moreover, the extracts of immunopositive clones induced puncta formation in N2A:APP_Swe_ cells not previously exposed to brain extracts (Olsson et al., 2018). Recent findings support that locally deposited Aβ species can result in both spread and propagation within the brain. Homogenates from the brains of AD patients and transgenic AD mouse models, but not wild type mice, when injected into the brains of transgenic AD mice resulted in Aβ pathology including amyloidosis, activation of microglia and astrocytes and neuritic dystrophy; the activity was blocked by Aβ immunodepletion (Langer et al., 2011). In view of the fact that AD and AD model brain-derived Aβ was far more potent than synthetic Aβ it was suggested that a specific conformation(s) created *in vivo* was important. Jucker and colleagues further characterized the active species showing that among the several fractions that harbored activity a soluble proteinase-K sensitive fraction was by far most potent (Langer et al., 2011). The ability for injected Aβ to induce both spreading and propagation was further examined by Prusiner and colleagues in experiments in mice transgenic for mutant APP and, to allow for live bioluminescence imaging, the luciferase gene driven by the GFAP promoter (i.e., TgAPP23:Gfap-luc mice). In these studies, injection of brain homogenates from two different transgenic AD models (TgAPP23; CRND8) resulted in marked increases in bioluminescence within about 250 days; there was no significant increase following injection of non-transgenic brain homogenate or in uninjected mice. At sacrifice there was a marked increase in Aβ levels and widespread deposition of Aβ within both the injected and non-injected hemispheres of those mice receiving transgenic inoculum. The results were replicated using a partially purified preparation of amyloid aggregates prepared from the same AD transgenic mice with the induction of an earlier increase in bioluminescence. Finally, injection of synthetic Aβ40 preparations also induced an increase in Aβ40 and Aβ42 levels and amyloid deposition. Importantly, the source of increased Aβ was the endogenously produced peptide because there was no evidence for the presence of synthetic Aβ at early times post inoculation (Stohr et al., 2012). Taken together with other findings, this study points strongly to the ability of AβOs, and possibly other Aβ-containing species, to engage in both spread and propagation. In so doing they lend support the idea that a prion-like mechanism contributes to the pathogenesis of AD (Langer et al., 2011; Stohr et al., 2012; Selkoe and Hardy, 2016).

Support for a prion mechanism for Aβ has recently been highlighted in studies on patients treated as children for short stature with Growth Hormone (GH) extracted from cadaveric human pituitaries (c-hGH), a practice abandoned with the availability of recombinant GH (r-hGH) in 1985. Such extracts were responsible for transmitting iatrogenically the prion disorder Creutzfeldt-Jakob disease (iCJD) with more than 200 cases to date and, given the long incubation period, more continuing to be documented (Jaunmuktane et al., 2015). Remarkably, an autopsy series of cases of iCJD of patients aged 36 to 51 demonstrated Aβ deposition in plaques and surrounding cortical and leptomeningeal vessels (CAA). The young age of these subjects and the absence of the *APOE*ε4 allele or for alleles for early onset AD or CAA were consistent with transmission and propagation of Aβ (Jaunmuktane et al., 2015). Importantly, very recently the same research team accessed archived c-hGH preparations of the type used to treat iCJD patients and showed that they contained Aβ x-40 and Aβ x-42, as well as tau. To test for the ability to seed Aβ pathology, intracerebral injections of c-hGH were made into a genetic knock-in model of AD in which the Aβ sequence was humanized and two AD mutations were introduced. Aβ deposits around cerebral vessels (i.e., CAA) and in cerebellum were present in mice inoculated with the archived GH preparations; such was not the case with r-hGH or if c-hGH was injected into wild type mice. Thus, the presence of humanized Aβ in the host was necessary for Aβ deposition (Purro et al., 2018). Pathological examination of a larger series of 27 iCJD cases confirmed the presence of CAA as the principal AD-related pathological signature. 21 of 27 cases showed widespread Aβ pathology, all of which showed CAA with about half also demonstrating plaques. There was no evidence for increased NFTs in cases with Aβ deposition, suggesting that Aβ deposition and tau pathology are distinct in terms of mechanism, timing, or both. In summary, the case for Aβ spread and propagation via a prion mechanism are increasingly well supported. As yet undefined are the mechanisms by which these events are initiated and sustained, the structure of Aβ species responsible, and the importance of spread and propagation for pathogenesis.

### Evidence for Soluble Toxic Tau Oligomers in Pathogenesis

The presence of tau pathology and its correlation with cognitive deficits encouraged the statement of the “tau hypothesis” of AD (Kametani and Hasegawa, 2018). As for the shift in focus from amyloid plaques to AβOs, the focus on tau-related pathology has moved from NFTs to tau oligomers. In support of this are observations pointing to lack of correlation between NFTs and synaptic dysfunction (Shafiei et al., 2017). In one such demonstration a P301S transgenic model showed hippocampal synaptic loss, hippocampal axonal spheroids and microglial activation 2 months before appearance of NFTs (Yoshiyama et al., 2007). In another, while aged mice expressing nonmutant human tau in the absence of mouse tau showed extensive neuron death, the latter was not directly correlated with tau filaments in degenerating neurons, suggesting that death occurs independently of NFT formation (Andorfer et al., 2005). In yet another study, it was possible to reduce tau overexpression in a mutant tau transgenic mice and reduce neuronal loss in spite of continued formation of NFTs (Santacruz et al., 2005). In a more recent investigation, the presence of NFTs in a mutant tau transgenic mouse was unrelated to the ability of visual experience to induce expression of Arc, an immediate-early gene linked to synaptic plasticity (Rudinskiy et al., 2014). These and other studies have substantiated the view that defects in axonal transport, synapse loss and other early signs of neurodegeneration are not linked to tau present in NFTs; however, NFTs may physically compromise intracellular transport and other cellular functions to contribute to later manifestations of disease (Ballatore et al., 2007; Brunden et al., 2008). Accordingly, the focus has shifted to other tau-containing species with recent work convincingly documenting a role for smaller assemblies, and in particular for tau oligomers (Shafiei et al., 2017).

Evidence pointing to the presence of tau oligomers in AD brain demonstrated isolated tau assemblies with a granular appearance. Ranging in size from 5 to 50 nm, the numbers of granular assemblies was markedly increased in the AD brain although, interestingly, not between early and advanced Braak stages (Maeda et al., 2006). Using a tau oligomer specific antibody raised against recombinant tau, Kayed and colleagues showed an approximate four-fold increase in the abundance of tau oligomers in the AD brain versus controls. Tau oligomers from the AD brain ranged in size from dimers to tetramers as observed by Western blotting. By immunostaining, tau oligomers were found to be present in pre-tangles, intraneuronal NFTs and neuropil threads and were also evident in the extracellular space. Using the same methods tau oligomers appeared to constitute a minority (20%) of total tau species in the AD brain, consistent with the view that tau oligomers are structurally distinct from other tau species and raising the possibility that the oligomeric conformation(s) contributes to toxic properties (Lasagna-Reeves et al., 2012b).

That tau oligomers are toxic has been demonstrated *in vitro* and *in vivo*. The AD brain serves as a source of potent tau oligomers. Isolated by immunoprecipitation using a tau oligomer-specific antibody, oligomers stable on SDS-PAGE migrated as dimers and trimers and by atomic force microscopy (AFM) the majority ranged in size from 4 to 8 nm. Added to hippocampal slices, isolated tau oligomers reduced LTP, an effect partially blocked by the antibody used to isolate them. When injected into wild type mice, but not tau knockout mice, tau oligomers reduced performance in the novel object recognition test of hippocampally mediated memory. Remarkably, months following injection there was widespread filamentous tau pathology and staining for hyperphosphorylated tau epitopes in the area of injection as well as neighboring regions. The latter stained negatively for human tau, illustrating the spread of tau pathology and a role for mouse tau in this process (Lasagna-Reeves et al., 2012a). In follow-on studies using the tau oligomer preparation from the AD brain, the Kayed group created a tau oligomer specific monoclonal antibody (TOMA) and used this to examine the emergence of tau pathology in a mouse expressing wild type human but not mouse tau. The levels of tau oligomers increased with age in these mice. In *in vitro* tests, TOMA blocked the death of neuroblastoma cells induced by AD brain-derived tau oligomers; remarkably viability was reduced at tau oligomer concentrations as low as 10 nM. *In vivo*, using the hippocampal cognitive test just referenced, intravenous TOMA injection was shown to block the effect of intracerebroventricular (ICV) injection of AD brain-derived tau oligomers, an effect that was correlated with reduced levels of tau oligomers in the injected mouse brain (Castillo-Carranza et al., 2014). Accordingly, tau oligomers do elicit toxic manifestations.

As for Aβ-mediated toxicity, important questions regarding tau oligomers are their source, structure, means of action, how they spread, and whether or not their spread benefits by a prion-like mechanism for templating and propagation. This active area of investigation has mapped the basic outline of the answers to these questions. First, tau is released as a full length protein in an activity-regulated manner from normal neurons *in vitro* (Pooler et al., 2013). The same applies to *in vivo* release in which the monomer is present in interstitial fluid (Yamada et al., 2014). Interestingly, the levels of monomer tau accessible via *in vivo* microdialysis were decreased following injection of aggregated tau, but not monomer tau, pointing to the ability *in vivo* of aggregated tau to modify the size and/or conformation of released endogenous tau (Yamada et al., 2011). Thus, in addition to presumed release of tau from dying cells, regulated release of tau and possibly tau oligomers may contribute to tau in brain interstitial fluid. Second, the evidence is strong that relatively small tau oligomers are toxic. *In vitro* studies point to low-n oligomers of tau, the smallest of which are trimers, as toxic at nM concentrations (Tian et al., 2013; Kaniyappan et al., 2017). While additional studies will be required to define toxic species, and will likely define a range of sizes and conformations of active species [e.g., see (Mirbaha et al., 2018) demonstrating a role for tau monomers], it is probable that a minimum size exists for a species that is able to be taken up by cells. Endocytosis, rather than cell membrane penetration, appears to serve as the means by which tau oligomers enter cells. Using HEK293 cells and neurons, Mirbaha and Colleagues showed that the trimer is minimum size for endocytosis of a recombinant tau oligomer as well as tau oligomers isolated from the AD brain (Mirbaha et al., 2015). Significantly, endocytosis of tau oligomers was dependent on cell surface heparin sulfate proteoglycans and endocytosis led to induction of intracellular tau aggregation, as assessed in HEK293 cells that expressed a split luciferase reporter to detect tau aggregation (Mirbaha et al., 2015). In separate studies, there was endocytic uptake of recombinant full length wild type tau, present as dimers and trimers or as short fibrils but not as monomers. After short incubation intervals endocytosed tau colocalized with a marker of bulk flow in Rab5 positive early endosomes and at later times was present in late endosomes and lysosomes. Uptake was demonstrated in both neuronal somas and axons and both anterograde and retrograde transport was documented (Wu et al., 2013). Using recombinant tau aggregates consisting of the microtubule binding region (MTBR), Diamond and colleagues showed that bulk flow endocytosis delivered MBTR aggregates to neural cells, resulting in displacement of tubulin. Using the same tau aggregates, treatment of either HEK293 cells or neural cells induced to express a full length wild type tau construct induced aggregation and fibril formation by the endogenously produced tau; remarkably, there was colocalization of exogenously added and endogenous tau proteins in intracellular inclusions, indicating direct interaction of added MBTR aggregates with endogenous tau (Frost et al., 2009).

The question as to whether or not tau spreads from cell to cell has been answered in the affirmative. In the series of studies just referenced, Frost and Colleagues showed that following 24 h co-culture of cells expressing differently labeled MTBRs a very small but significant percent of cells showed colocalization of the two proteins, pointing to cell-to-cell transfer of tau (Frost et al., 2009). Several physiologically relevant models have demonstrated robust spread of tau species. In one, Hyman and colleagues using microfluidic cultures that allow for fluidic isolation of neuron cell bodies and their processes demonstrated neuron to neuron transfer of a PBS-soluble tau species isolated from a mouse expressing a mutant tau transgene, the rTg4510 mouse. Interestingly, evidence was provided that the tau species most efficiently endocytosed was globular, immunoreactive with a tau oligomer specific antibody and highly phosphorylated; it migrated on size exclusion chromatography in the HMW range (>670 kD) (Takeda et al., 2015). Endocytosed tau oligomers were colocalized with markers of the Golgi apparatus and even more robustly with lysosomes. Tau uptake and transneuronal spread was blocked by antibodies to tau (Nobuhara et al., 2017). *In vivo*, HMW tau species injected into rTg4510 and wild type mice were also taken up by neurons. Importantly, the interstitial fluid of rTg4510 mice was shown to contain tau species in the HMW range as well as intermediate and low molecular weights and this source of tau also supported endocytosis by neurons. PBS-soluble tau oligomers isolated from the AD brain showed similar characteristics, as well as uptake and transneuronal spread in microfluidic chambers (Takeda et al., 2015).

The ability of tau to spread *in vivo* has been dramatically demonstrated in studies in which viral delivery of wild type human tau to hippocampus or transgene driven expression of mutant tau in entorhinal cortex was followed by the appearance of human tau in neuron cell bodies first in neighboring and then in distant brain regions to which the transgene expressing cells were connected. Transcellular transmission was convincingly demonstrated and spread was marked by induction of pathological tau staining (i.e., hyper-phosphorylated epitopes, Gallyas staining and positivity for Thioflavin-S) and by co-aggregation of human and endogenous mouse tau (de Calignon et al., 2012; Liu et al., 2012; Dujardin et al., 2014). These studies point to tau spread, but evidence of increasing tau pathology and of the interaction between human and mouse tau in locations distant from the site of human tau expression also supports tau as able to seed and propagate pathology. Diamond and colleagues have provided compelling evidence to support a prion-like mechanism by which abnormal tau isoforms template normal isoforms to induce propagation of aberrant tau species. Distinct tau strains, created by treating HEK293 cells with fibrils of the recombinant repeat domain of tau (tau-RD), demonstrated specific patterns for immunostaining and biochemical properties. Strain characteristics were stably transmitted *in vitro* and strain-specific tau inclusions were present when strain-containing lysates were transferred to naïve tau-RD expressing cells as well as to primary neurons. Inoculation of the brain of P301S mutant tau mice with strain lysates also induced strain-specific tau inclusions. Strains were also stably propagated *in vivo* over several generations. When tau immunoisolated from brain-passaged strains were used to inoculate the cells used to create strains the original morphological and biochemical properties were recapitulated (Sanders et al., 2014). Recent studies have built upon these findings to provide evidence for the existence of several tau strains and sub-strains that dictate distinct pathological phenotypes and rates of progression. Evidence that different tauopathies, one of which is AD, harbor different tau strains points to a role for strain-specific structures in defining disease pathogenesis (Sanders et al., 2014; Sharma et al., 2018). Indeed, taken together, the data powerfully inform the biology of tau in AD pathogenesis, pointing to the presence of transneuronal spread and a prion-like process for templating and propagation of tau pathology.

Important unanswered questions concern the how endocytosed tau oligomers gain access to cytosol, by what mechanism(s) they act to induce toxicity, the structures of seed-competent and toxic species, and the extent to which such species are accessible to therapeutic approaches, including antibodies. The importance of addressing treatment options is now informed by the evidence that tau spread may occur via membrane-free tau species, as supported above (Nobuhara et al., 2017) as well as by exosomes and ectosomes (Asai et al., 2015; Shafiei et al., 2017). Also important is the question as to how the actions of toxic AβOs and tau interact and possibly reinforce one another. Evidence that total Aβ and prefibrillar AβOs are present in relatively larger amounts than p-tau in parietal cortex synaptosomes in early stage AD samples prepared from the parietal cortex suggests that Aβ species precede those for p-tau in synaptic domains. Interestingly, synaptosomes sorted for Aβ were more likely to be positive for p-tau than total synaptosomes, suggesting the Aβ may increase synaptic p-tau (Bilousova et al., 2016), a suggestion consistent with other studies demonstrating the ability of Aβ preparations to induce cellular p-tau (Zheng et al., 2002; Jin et al., 2011). Additional support for a primary role for AβOs in pathogenesis is that in FAD and DS Aβ pathology emerges prior to the accumulation of NFTs. In contrast, mutations in tau lead to a number of progressive neurodegenerative disorders in which there is no accumulation of Aβ (Selkoe and Hardy, 2016). Animal studies also support the primacy of Aβ. In one case, tau pathology in mice transgenic for tau was increased by crossing with APP transgenic mice without an increase in Aβ deposition (Lewis et al., 2001). Equally important are studies that point to an interaction(s) between Aβ and tau in creating AD-relevant phenotypes (Selkoe and Hardy, 2016). In a very recent study Hyman and colleagues examined neuronal activity in the APPSwe/PS1ΔE9 and rTg4510 mutant tau (P301L) transgenic mice. While APPSwe/PS1ΔE9 mice showed cortical neuronal hyperactivity, in rTg4510 mice activity was suppressed. Remarkably, crossing the two mouse lines resulted in a marked reduction in neuronal activity, even in very young mice before the onset of overt neuropathology (Busche et al., 2019). We conclude that there is much to learn about the separate activities induced by Aβ- and tau-linked pathological processes, not the least of which is crosstalk between these species and the pathogenetic events they induce.

### Synthesis and Speculations on AD Pathogenesis

The data reviewed above point to key features of the pathology and genetics of AD that have informed our view of pathogenesis. However, important questions remain as to how these findings allow for a cogent synthesis of AD pathogenesis. Key unanswered questions include: (1) the disconnect between the initial locus, timing and regional spread of amyloid and tau pathology, as reflected in the presence of amyloid plaques and NFTs. Why, if Aβ initiates the pathogenetic cascade does the time of emergence and locus of these pathologies differ? (2) the correlation of NFTs but not amyloid plaques with cognitive dysfunction. How does one explain the far more convincing link between tau pathology and dementia? (3) the evidence that synaptic and neuronal loss are better correlated with loss of cognition than NFTs. If there are molecular and cellular processes that link tau pathology to synaptic dysfunction, what role is played by NFTs? (4) the role played by early endosomes, whose involvement marked by increased size serves as a pathological marker that precedes the presence of plaques and NFTs. How could changes in early endosomes impact the ability of neurons to function and contribute to pathogenesis? (5) the selective vulnerability of neurons, including the locus coeruleus, basal forebrain cholinergic complex and raphe as well as entorhinal cortex layer II neurons. What factor(s) predispose these neurons to AD pathogenesis? (6) the impact of genetic and environmental risk factors on the onset and progression of AD. In particular, how does the genetic evidence inform pathogenesis?

**FIGURE 2:**
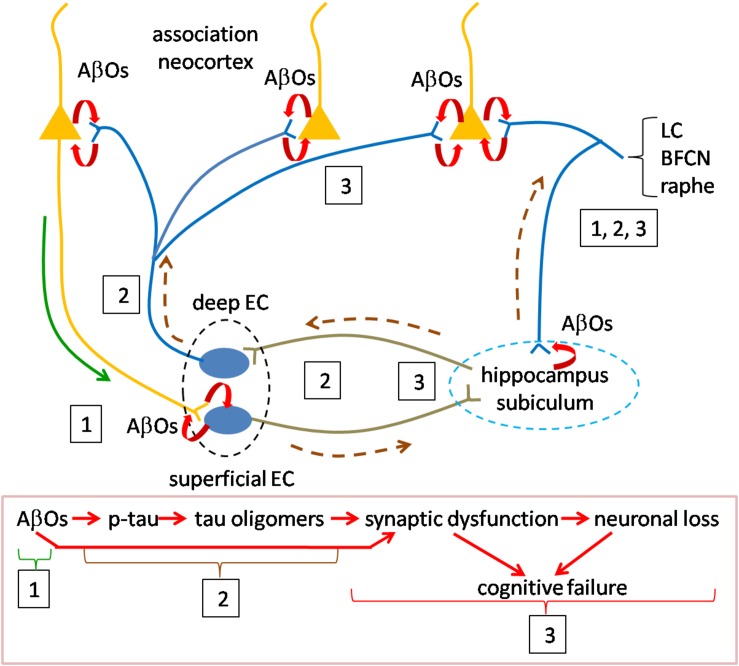
Speculative scheme to explain AD pathogenesis. Overview of pathogenesis is summarized in the red boxed caption at the bottom. Initiating pathogenesis: The process would be initiated by the formation of AβOs in cortical neurons. The source of AβOs would include those produced locally within the presynaptic terminals of cortical neurons innervating entorhinal cortex. AβOs produced in the somatodendritic domain of cortical neurons could serve as the nidus for local amyloid plaque deposition and would be in contact with axons of entorhinal cortical neurons. Thus, AβOs produced in both the cortico-entorhinal and entorhinal-cortical projections may participate in formation of AβOs, The initial focus of AβO actions (signified by boxed number 1) may be registered in superficial entorhinal cortex whose neurons receive cortical inputs. At later stages retrograde AβOs acting on entorhinal axons in cortex may contribute to pathogenesis, as may AβOs produced in entorhinal afferents to cortex (boxed 3). Driving pathogenesis: AβO acting on entorhinal neurons would catalyze formation of tau oligomers with subsequent spread and propagation in connected neurons, first in hippocampus and subiculum and then association neocortex (boxed 2). Toxic tau oligomers could exert injury to the neurons in which they are formed and following release to the synapses and neurons to which they spread and in which they propagate (boxed 3). AβOs could interact during this phase with tau oligomers to enhance synaptic and neuronal dysfunction. Neurons in the locus coeruleus, basal forebrain cholinergic complex and raphe nuclei may owe their selective vulnerability to their connections with both neocortex and hippocampus during early and later phases (boxed 1, 2, 3). Curved red arrows signify local production of AOs due to processing of APP. Green solid line (near boxed 1) indicates the anterograde transport of APP for processing at the presynapse during the initial phase; AβOs produced in somatodendritic domains could also be transported anterogradely at this stage (green line). Brown dashed line represents the direction of tau oligomer spread. LC, locus coeruleus neuron; BFCN, basal forebrain cholinergic neuron; and EC, entorhinal cortex neuron.

We think that the discoveries related to the presence and activity of oligomers of Aβ and tau, reviewed above provide key insights for answering these questions. We offer a speculative synthesis of the events underlying the initiation and progression of AD pathogenesis, as summarized in Figure 2. The fundamental assertion is that AβOs and tau oligomers activate the causal mechanisms for AD pathogenesis and that they are responsible for the emergence of all of the pathological markers of AD. Our synthesis focuses on the distribution and actions of oligomers in inducing AD.

#### Initiating Pathogenesis via AβOs

The process would be initiated by the formation of AβOs produced by cortical neurons, including those located in basal frontal, temporal and occipital regions. An unanswered question is how Aβ species normally present in these neurons becomes toxic. Possible causes include one or more of the following: an increase in the levels of Aβ species, in particular Aβ42; the new presence of oligomeric assemblies; and a change in the structure of oligomers that confers toxicity and/or the ability to spread and/or propagate. Possibly relevant to such events are the genetic evidence for increased risk due to the ε4 allele of *APOE*. For example, reduced Aβ clearance due to the ε4 allele (Shinohara et al., 2013; Mulder et al., 2014) may contribute directly or indirectly to increased AβO levels. Other risk alleles that could contribute to changes in APP processing and AβO levels are those for genes whose products regulate lipid metabolism (Di Paolo and Kim, 2011). Whatever the underlying mechanism for increased AβOs, their production in cortical neurons would initiate the pathogenetic cascade. AβOs produced in the somatodendritic domain of cortical neurons could serve as the nidus for local amyloid plaque deposition. More important to pathogenesis, and likely significantly proceeding amyloid plaque accumulation, AβOs produced by cortical neurons would gain access to neurons of the entorhinal cortex. This likely occurs in the presynaptic terminals of cortical axons through local processing of APP followed by AβO, but might also be supported by anterograde vesicular axonal transport of AβOs to entorhinal cortex. It is noteworthy that the superficial layers of the transentorhinal and entorhinal region receive a massive input from cortex and that these regions, in turn, convey information to hippocampus (Witter et al., 2017). Moreover, the entorhinal cortex receives hippocampal afferents and projects to neocortex where their axons engage cortical neurons and would thereby be exposed to this source of AβOs. Local production of AβOs in entorhinal cortex neurons themselves, possibly stimulated by cortical inputs, may also play a role at this early stage of pathogenesis (Gouras et al., 2000; Kobro-Flatmoen et al., 2016). Also at this stage, early endosomal processing of APP may prove critical (Haass et al., 2012). The enlargement of early endosomes and dysregulation of their function, with subsequent disruption of the endosomal and lysosomal pathways (Nixon, 2017; Chen et al., 2018) could serve to both mediate and amplify local AβO-mediated events. In one example, activation of Rab5 could result in increased endocytosis of surface receptors for neurotransmitters and neurotrophic factors thus compromising synaptic signaling and trophic support (Chen et al., 2018). In support of a role for endosomes GWAS studies have identified risk variants in a number of genes whose products serve in the endocytosis pathway (Karch and Goate, 2015).

#### Driving Pathogenesis via Tau Oligomers

AβOs acting in entorhinal cortex would catalyze the next stage of pathogenesis in which tau oligomers formed within entorhinal cortical neurons would spread to connected neurons, first in hippocampus and then cortex. These events would be mediated, at least in part, by anterograde transport of tau oligomers and would be supported by propagation of tau species in connected neurons. How this process is initiated and driven is yet to be defined; both cortically derived and locally produced AβOs may play a role (Harris et al., 2010). Support for this synthesis comes from studies cited above and additional findings. The first is that Aβ has been shown to induce phosphorylation of tau (Zheng et al., 2002; Stoothoff and Johnson, 2005). Unknown is whether or not Aβ-mediated tau phosphorylation leads to production of tau oligomers, but this is feasible. Increased levels of tau, changes in tau phosphorylation, and the emergence of novel, toxic and mobile oligomeric species may each contribute. In this regard the evidence that amyloidosis increases tau synthesis may be highly instructive (Sato et al., 2018). Nevertheless, we regard the mechanistic link(s) between AβOs and tau oligomers as a critical missing piece in understanding AD pathogenesis.

The second support is evidence for the spread and propagation of tau oligomers. As demonstrated in animal and cellular models, the data is compelling that tau pathology spreads anterogradely from entorhinal cortex to nearby and distant neurons. It is plausible that the emergence of tau pathology as reflected in pre-tangles and NFTs can serve as a guide to the pattern of spread. Indeed, it is reasonable to suggest that tau oligomers serve as the precursors for pre-tangles and NFTs. Accepting this, the pattern of spread corresponding to Braak staging, reviewed above, is highlighted by initial involvement of transentorhinal/entorhinal cortex, then hippocampus and temporal cortex followed by subiculum and presubiculum and only later association neocortex. Tau PET staging largely confirms this pattern for spread of tau pathology. It is interesting that increasing Aβ burden, assessed using amyloid PET imaging, predicts spread of tau pathology, also assessed by PET, beyond medial temporal cortex to lateral and inferior temporal cortex as well as other neocortical regions (Scholl et al., 2016). This finding raises the question as to how Aβ- and tau-mediated pathologies interact and possibly reinforce one another.

A third key point is that tau oligomers are toxic. Thus, tau pathology would be linked to injury to the neurons that harbor tau oligomers and to the synapses and neurons to which they are spread. Indeed, as addressed above, changes in cognitive function are most closely correlated with both tau pathology and synaptic and neuronal loss. Here again, the interaction of AβOs and tau oligomers may prove important and even synergistic (Spires-Jones and Hyman, 2014).

Special mention is deserved for those neuronal populations shown to be highly vulnerable in AD. While there is much to learn about the underlying mechanisms, we note two features that may contribute. The first is that neurons of the locus coeruleus, basal forebrain cholinergic complex and raphe innervate wide cortical territories (Avery and Krichmar, 2017). Therefore, like entorhinal cortex, they would be exposed to the AβOs produced by cortical neurons. Second, they project to the hippocampus proper, thus possibly exposing their axons to the AβOs and tau oligomers present in this early target of AD pathogenesis. These and other mechanisms may be responsible for early pathological manifestations in these neurons and their marked loss in AD. Why tau pathology so prominently impacts entorhinal and transentorhinal cortex in early AD is unknown, but one can speculate that higher levels of AβOs and perhaps other factors intrinsic to these neurons may contribute.

#### Inflammation Enhancing Pathogenesis

The role that inflammatory mechanisms play in pathogenesis has only recently been given the attention deserved. Genetic evidence for the involvement of microglial-expressed proteins has increased interest and microglial activation and participation in the phagocytosis of Aβ and production of cytokines is now viewed as significant (Sarlus and Heneka, 2017). How the products of neuronal injury contribute to the inflammatory milieu needs further definition. Indeed, though there is much to learn, inflammatory events, especially those involving microglia, may be poised to amplify the pathogenetic mechanisms engaged by AβOs and tau oligomers.

## Summary, Suggestions, and Conclusion

More than 100 years after Alois Alzheimer report of a patient whose disorder now bears his name, and in spite of detailed neuropathological studies and 40 years of robust research investments in AD, there is much to learn about pathogenesis and the need for disease-modifying treatments continues. Nevertheless, findings from genetic, molecular and cellular studies are giving unprecedented insights that have changed our concepts of pathogenesis, thus hastening the pace of discovery of disease mechanisms and identification of therapeutic targets. We view studies of the structure and function of oligomeric species of Aβ and tau as among the most compelling for understanding pathogenesis and defining treatments and offer a speculative synthesis for how their actions both initiate and drive pathogenesis.

Elucidating pathogenesis will benefit by the application of new research tools. A key issue facing such studies and those for drug discovery in AD has been the need for model systems that recapitulate the pathological signatures of the disease as well as the molecular mechanisms that precipitate the disorder. Human pluripotent stem cell-based models, which convert fibroblasts and certain other cell types (i.e., fibroblasts, blood cells, keratinocytes, etc.) through a pluripotent stem cell stage (i.e., induced pluripotent stem cell: iPSC) to neurons and other brain cell types (e.g., astrocytes, microglial cells), are increasingly used in research in AD and other brain disorders (Zeltner and Studer, 2015; Mertens et al., 2018). The use of a variety of cell types, ability to generate cells in very large numbers, robust differentiation paradigms and gene editing tools to create isogenic lines have greatly enhanced the value of iPSCs for modeling childhood and monogenetic diseases. However, most cases of AD are sporadic, occur in the geriatric population and do not evidence a strong genetic component. Recent technological advances have made it possible to directly induce neurons from fibroblasts (induced neurons: iNs). Unlike iPSCs, iNs preserve signatures of cellular aging and reflect an adult-like neuronal identity, thus qualifying them as uniquely suited to studies of LOAD (Huh et al., 2016; Tang et al., 2017; Kim et al., 2018; Mertens et al., 2018; Victor et al., 2018). A limitation of the iN approach is that, unlike iPSC there is no highly expandable intermediate stage, thus reducing the number of neurons available for study (Mertens et al., 2018). Additionally, systems level analysis of genetic and epigenetic changes in neurons derived from AD fibroblasts using iPSC and iN methods will likely contribute. Indeed, integrating RNA-Seq, ATAC-Seq, and ChIP-Seq approaches promises to create a wealth of new data and new insights into the biology of AD (Caldwell et al., 2019). Finally, the use of these cells to make human cerebroids now makes it possible to examine reprogrammed cells from patients and controls in brain-like cellular structures *in vitro* and *in vivo* (Lancaster and Knoblich, 2014), and to test possible treatments in these more realistic and yet fully human contexts. In conclusion, we argue that the future holds great promise for intercepting the epidemic of AD.

## Author Contributions

X-QC and WCM wrote and edited the manuscript, and approved it for publication.

## Conflict of Interest Statement

The authors declare that the research was conducted in the absence of any commercial or financial relationships that could be construed as a potential conflict of interest.
